# 3-Hy­droxy-1-(4-meth­oxy­benz­yl)piperidin-2-one

**DOI:** 10.1107/S1600536812049501

**Published:** 2012-12-08

**Authors:** Daniel P. Pienaar, Sanaz Khorasani, Charles B. de Koning, Joseph P. Michael

**Affiliations:** a Molecular Sciences Institute, School of Chemistry, University of the Witwatersrand, PO Wits 2050, Johannesburg, South Africa

## Abstract

The title compound, C_13_H_17_NO_3_, adopts a conformation in which the aromatic ring and the mean plane of the piperidine ring are almost perpendicular to each other [dihedral angle = 79.25 (6)°]. The presence of the carbonyl group alters the conformation of the piperidine ring from a chair to a twisted half-chair conformation. In the crystal, pairs of strong O—H⋯O hydrogen bonds link the mol­ecules into inversion dimers. Weak C—H⋯O inter­actions extend the hydrogen-bonding network into three dimensions.

## Related literature

For the use of related lactams in the synthesis of febrifugine analogues, see: Michael *et al.* (2006 ▶). For information on the biological activity of febrifugine, a quinazoline alkaloid with potent anti­malarial activity, see: Murata *et al.* (1998 ▶). For the use of chiral oxaziridines in asymmetric hy­droxy­lation, see: Davis *et al.* (1990 ▶). For the conformation of six-membered rings, see: Boeyens (1978 ▶).
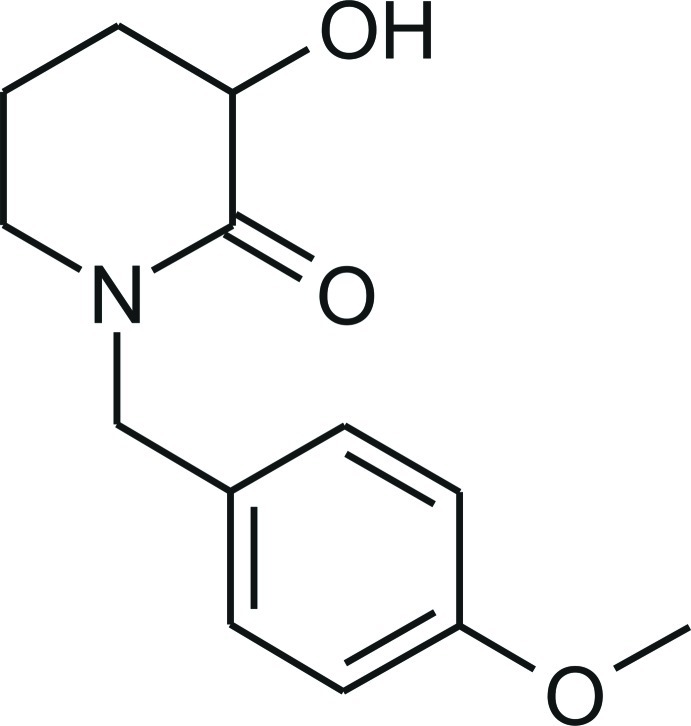


## Experimental

### 

#### Crystal data

C_13_H_17_NO_3_
*M* *_r_* = 235.28
Monoclinic, 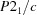

*a* = 12.980 (3) Å
*b* = 7.6143 (17) Å
*c* = 12.189 (3) Å
β = 90.497 (5)°
*V* = 1204.6 (5) Å^3^
*Z* = 4
Mo *K*α radiation
μ = 0.09 mm^−1^
*T* = 173 K
0.32 × 0.26 × 0.18 mm

#### Data collection

Bruker APEXII CCD diffractometer
8378 measured reflections
2895 independent reflections
2271 reflections with *I* > 2σ(*I*)
*R*_int_ = 0.027

#### Refinement

*R*[*F* ^2^ > 2σ(*F* ^2^)] = 0.042
*wR*(*F* ^2^) = 0.124
*S* = 1.08
2895 reflections
158 parameters
H atoms treated by a mixture of independent and constrained refinement
Δρ_max_ = 0.52 e Å^−3^
Δρ_min_ = −0.22 e Å^−3^

### 

Data collection: *APEX2* (Bruker, 2005 ▶); cell refinement: *SAINT-NT* (Bruker, 2005 ▶); data reduction: *SAINT-NT*; program(s) used to solve structure: *SHELXS97* (Sheldrick, 2008 ▶); program(s) used to refine structure: *SHELXL97* (Sheldrick, 2008 ▶); molecular graphics: *ORTEP-3 for Windows* (Farrugia, 2012 ▶) and *SCHAKAL99* (Keller, 1999 ▶); software used to prepare material for publication: *WinGX* (Farrugia, 2012 ▶) and *PLATON* (Spek, 2009 ▶).

## Supplementary Material

Crystal structure: contains datablock(s) global, I. DOI: 10.1107/S1600536812049501/bh2468sup1.cif

Structure factors: contains datablock(s) I. DOI: 10.1107/S1600536812049501/bh2468Isup2.hkl

Additional supplementary materials:  crystallographic information; 3D view; checkCIF report

## Figures and Tables

**Table 1:** Hydrogen-bond geometry (Å, °)

*D*—H⋯*A*	*D*—H	H⋯*A*	*D*⋯*A*	*D*—H⋯*A*
O2—H2⋯O1^i^	0.96 (2)	1.84 (2)	2.7708 (16)	161.6 (19)
C6—H6*B*⋯O1^ii^	0.99	2.43	3.3142 (17)	148
C14—H14*B*⋯O2^iii^	0.98	2.52	3.449 (2)	158

Symmetry codes: (i) 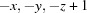; (ii) 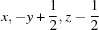; (iii) 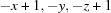.

**Fig. 1.**
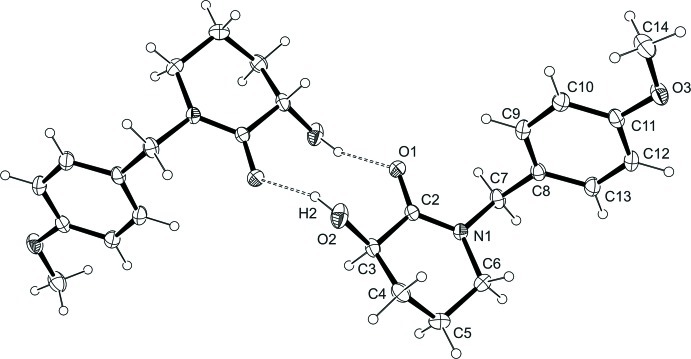
The molecular structure of the title compound, showing the hydrogen bonding to another molecule related by a center of inversion. Displacement ellipsoids are drawn at the 50% probability level.

**Fig. 2.**
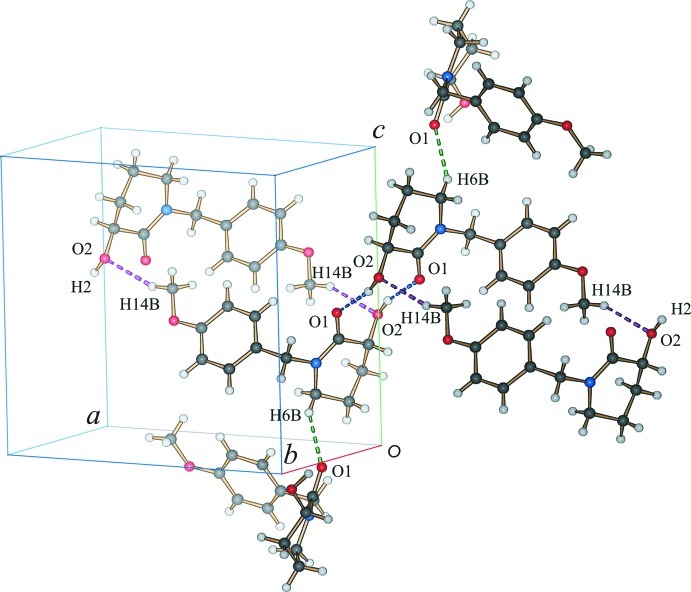
O—H···O and C—H···O interactions in the crystal structure of the title compound, which result in an extensive hydrogen bonding network in three dimensions.

## supplementary crystallographic information

### Comment

The title piperidinone was prepared as an early intermediate for the total synthesis of febrifugine, a quinazoline alkaloid with potent antimalarial activity (Murata *et al.*, 1998). Ongoing investigations in our laboratories have made use of similar lactams for the synthesis of febrifugine analogues (Michael *et al.*, 2006). It should be noted that, although the 3-hydroxy substituent was introduced by attempted asymmetric hydroxylation of the enolate of 1-(4-methoxybenzyl)piperidin-2-one with (+)-camphorsulfonyloxaziridine (Davis *et al.*, 1990), partial racemization occurred; the crystals selected for analysis proved to be racemic.

The title organic compound (Fig. 1) adopts a conformation in which the aromatic ring and the piperidine ring are almost perpendicular to each other. Ring puckering analysis, as implemented in *PLATON* (Spek, 2009), indicates that the piperidine ring adopts a twisted half-chair conformation owing to the presence of the carbonyl group (Boeyens, 1978). Several hydrogen bonds exist in the structure (Table 1), with the most significant being an O—H···O hydrogen bond. These result in the formation of hydrogen bonded pairs of molecules which are related to each other by a center of inversion (Fig. 1). These molecules interact further through C—H···O interactions (Fig. 2) resulting in an extensive hydrogen bonding network of molecules.

### Experimental

To a solution of lithium hexamethyldisilazide, prepared from *n*-butyllithium (1.6 *M* in hexane, 1.83 ml, 2.93 mmol) and hexamethyldisilazane (0.63 ml) in THF (10 ml) at -70 °C was added a solution of 1-(4-methoxybenzyl)piperidin-2-one (322 mg, 1.47 mmol) in THF (20 ml). The solution was stirred at this temperature for 1 h, after which a solution of (+)-camphorsulfonyloxaziridine (0.67 g, 2.9 mmol) in THF (20 ml) was added dropwise. Stirring was maintained for a further 16 h at temperatures kept between -70 and -60 °C. The reaction was quenched by addition of saturated aqueous ammonium chloride solution (10 ml) and allowed to warm to ambient temperature. The organic components were extracted with dichloromethane (4 × 15 ml), the combined organic layers were washed with brine (20 ml), dried over MgSO_4_, and concentrated *in vacuo*. Purification by column chromatography on silica gel with hexane-ethyl acetate mixtures (9:1 to 1:1 *v*/*v*) yielded the title compound, which was recrystallized from hexane-ethyl acetate to yield the product as irregularly shaped colourless crystals (261 mg, 75%), m.p. 347–349 K.

### Refinement

All H atoms attached to C atoms were positioned geometrically, and allowed to ride on their parent atoms, with C—H bond lengths of 0.95 Å (Ar—H), 1.0 (CH), 0.99 Å (CH_2_) or 0.98 Å (CH_3_), and isotropic displacement parameters set to 1.2 (CH and CH_2_) or 1.5 times (CH_3_) the *U*_eq_ of the parent atom. The alcohol H atom (H2) was located from the difference map and refined freely with isotropic displacement parameter set to 1.5 times the *U*_eq_ of the parent atom O2.

### Crystal data

**Table:**

C_13_H_17_NO_3_	*F*(000) = 504
*M**_r_* = 235.28	*D*_x_ = 1.297 Mg m^−^^3^
Monoclinic, *P*2_1_/*c*	Melting point: 347 K
Hall symbol: -P 2ybc	Mo *K*α radiation, λ = 0.71073 Å
*a* = 12.980 (3) Å	Cell parameters from 958 reflections
*b* = 7.6143 (17) Å	θ = 3.5–28.3°
*c* = 12.189 (3) Å	µ = 0.09 mm^−^^1^
β = 90.497 (5)°	*T* = 173 K
*V* = 1204.6 (5) Å^3^	Irregular, colourless
*Z* = 4	0.32 × 0.26 × 0.18 mm

### Data collection

**Table:**

Bruker APEXII CCD diffractometer	2271 reflections with *I* > 2σ(*I*)
Radiation source: fine-focus sealed tube	*R*_int_ = 0.027
Graphite monochromator	θ_max_ = 28.0°, θ_min_ = 3.1°
φ and ω scans	*h* = −14→17
8378 measured reflections	*k* = −10→10
2895 independent reflections	*l* = −16→9

### Refinement

**Table:**

Refinement on *F*^2^	Primary atom site location: structure-invariant direct methods
Least-squares matrix: full	Secondary atom site location: difference Fourier map
*R*[*F*^2^ > 2σ(*F*^2^)] = 0.042	Hydrogen site location: inferred from neighbouring sites
*wR*(*F*^2^) = 0.124	H atoms treated by a mixture of independent and constrained refinement
*S* = 1.08	*w* = 1/[σ^2^(*F*_o_^2^) + (0.066*P*)^2^ + 0.1896*P*] where *P* = (*F*_o_^2^ + 2*F*_c_^2^)/3
2895 reflections	(Δ/σ)_max_ < 0.001
158 parameters	Δρ_max_ = 0.52 e Å^−^^3^
0 restraints	Δρ_min_ = −0.22 e Å^−^^3^
0 constraints	

### Fractional atomic coordinates and isotropic or equivalent isotropic displacement parameters (Å^2^)

**Table:**

	*x*	*y*	*z*	*U*_iso_*/*U*_eq_	
C2	0.11028 (9)	0.10275 (17)	0.35921 (10)	0.0283 (3)	
C3	0.06871 (9)	−0.07971 (17)	0.32619 (11)	0.0304 (3)	
H3	−0.0060	−0.0662	0.3072	0.037*	
C4	0.12221 (11)	−0.15543 (17)	0.22690 (12)	0.0353 (3)	
H4A	0.0856	−0.2621	0.2013	0.042*	
H4B	0.1939	−0.1883	0.2463	0.042*	
C5	0.12221 (11)	−0.01793 (18)	0.13695 (11)	0.0374 (3)	
H5A	0.1507	−0.0688	0.0689	0.045*	
H5B	0.0508	0.0209	0.1214	0.045*	
C6	0.18684 (10)	0.13727 (17)	0.17295 (10)	0.0323 (3)	
H6A	0.2605	0.1038	0.1716	0.039*	
H6B	0.1766	0.2349	0.1204	0.039*	
C7	0.20555 (10)	0.36915 (17)	0.31491 (12)	0.0334 (3)	
H7A	0.1693	0.4153	0.3800	0.040*	
H7B	0.1951	0.4535	0.2540	0.040*	
C8	0.31965 (10)	0.35530 (15)	0.34078 (11)	0.0296 (3)	
C9	0.35322 (10)	0.27078 (17)	0.43529 (11)	0.0343 (3)	
H9	0.3037	0.2227	0.4838	0.041*	
C10	0.45748 (11)	0.25429 (18)	0.46121 (11)	0.0349 (3)	
H10	0.4786	0.1967	0.5268	0.042*	
C11	0.53038 (10)	0.32307 (16)	0.39013 (11)	0.0321 (3)	
C12	0.49838 (10)	0.4107 (2)	0.29565 (12)	0.0389 (3)	
H12	0.5479	0.4600	0.2477	0.047*	
C13	0.39415 (10)	0.42600 (19)	0.27143 (11)	0.0365 (3)	
H13	0.3730	0.4856	0.2066	0.044*	
C14	0.66904 (13)	0.2242 (2)	0.50439 (15)	0.0495 (4)	
H14A	0.6450	0.2894	0.5686	0.074*	
H14B	0.7445	0.2192	0.5055	0.074*	
H14C	0.6411	0.1047	0.5061	0.074*	
N1	0.16062 (8)	0.19805 (13)	0.28388 (8)	0.0270 (2)	
O1	0.09468 (8)	0.15790 (15)	0.45317 (8)	0.0444 (3)	
O2	0.07623 (8)	−0.19755 (14)	0.41449 (10)	0.0457 (3)	
H2	0.0256 (18)	−0.165 (3)	0.4675 (17)	0.069*	
O3	0.63479 (7)	0.31060 (14)	0.40678 (9)	0.0431 (3)	

### Atomic displacement parameters (Å^2^)

**Table:**

	*U*^11^	*U*^22^	*U*^33^	*U*^12^	*U*^13^	*U*^23^
C2	0.0217 (5)	0.0342 (6)	0.0289 (6)	−0.0031 (5)	−0.0005 (4)	0.0000 (5)
C3	0.0229 (6)	0.0295 (6)	0.0389 (7)	−0.0030 (5)	−0.0027 (5)	0.0049 (5)
C4	0.0332 (7)	0.0276 (6)	0.0451 (8)	0.0003 (5)	−0.0046 (6)	−0.0036 (5)
C5	0.0420 (8)	0.0393 (7)	0.0309 (7)	0.0059 (6)	−0.0042 (5)	−0.0062 (5)
C6	0.0340 (7)	0.0356 (7)	0.0272 (6)	0.0037 (5)	0.0051 (5)	0.0026 (5)
C7	0.0288 (6)	0.0249 (6)	0.0465 (8)	−0.0013 (5)	0.0043 (5)	−0.0014 (5)
C8	0.0284 (6)	0.0234 (6)	0.0371 (7)	−0.0034 (5)	0.0042 (5)	−0.0038 (5)
C9	0.0309 (7)	0.0336 (7)	0.0385 (7)	−0.0038 (5)	0.0097 (5)	0.0021 (5)
C10	0.0352 (7)	0.0348 (7)	0.0349 (7)	−0.0028 (5)	0.0019 (5)	0.0031 (5)
C11	0.0268 (6)	0.0293 (6)	0.0404 (7)	−0.0052 (5)	0.0016 (5)	−0.0049 (5)
C12	0.0318 (7)	0.0448 (8)	0.0402 (7)	−0.0106 (6)	0.0075 (5)	0.0056 (6)
C13	0.0343 (7)	0.0377 (7)	0.0377 (7)	−0.0067 (6)	0.0023 (5)	0.0067 (6)
C14	0.0375 (8)	0.0456 (9)	0.0652 (11)	−0.0059 (7)	−0.0124 (7)	0.0064 (7)
N1	0.0249 (5)	0.0262 (5)	0.0300 (5)	−0.0014 (4)	0.0030 (4)	0.0001 (4)
O1	0.0430 (6)	0.0598 (7)	0.0307 (5)	−0.0174 (5)	0.0094 (4)	−0.0094 (5)
O2	0.0365 (6)	0.0453 (6)	0.0553 (7)	0.0001 (4)	0.0051 (5)	0.0202 (5)
O3	0.0268 (5)	0.0473 (6)	0.0553 (7)	−0.0067 (4)	−0.0021 (4)	0.0046 (5)

### Geometric parameters (Å, º)

**Table:**

C2—O1	1.2381 (15)	C7—H7B	0.9900
C2—N1	1.3443 (16)	C8—C9	1.3865 (19)
C2—C3	1.5426 (18)	C8—C13	1.3977 (18)
C3—O2	1.4039 (16)	C9—C10	1.393 (2)
C3—C4	1.5145 (19)	C9—H9	0.9500
C3—H3	1.0000	C10—C11	1.3909 (19)
C4—C5	1.5160 (19)	C10—H10	0.9500
C4—H4A	0.9900	C11—O3	1.3719 (16)
C4—H4B	0.9900	C11—C12	1.391 (2)
C5—C6	1.5121 (19)	C12—C13	1.3871 (19)
C5—H5A	0.9900	C12—H12	0.9500
C5—H5B	0.9900	C13—H13	0.9500
C6—N1	1.4717 (16)	C14—O3	1.4272 (19)
C6—H6A	0.9900	C14—H14A	0.9800
C6—H6B	0.9900	C14—H14B	0.9800
C7—N1	1.4753 (16)	C14—H14C	0.9800
C7—C8	1.5154 (18)	O2—H2	0.96 (2)
C7—H7A	0.9900		
			
O1—C2—N1	122.21 (12)	C8—C7—H7B	109.2
O1—C2—C3	119.16 (11)	H7A—C7—H7B	107.9
N1—C2—C3	118.62 (11)	C9—C8—C13	117.85 (12)
O2—C3—C4	109.87 (11)	C9—C8—C7	120.32 (11)
O2—C3—C2	110.71 (11)	C13—C8—C7	121.84 (12)
C4—C3—C2	112.93 (10)	C8—C9—C10	121.88 (12)
O2—C3—H3	107.7	C8—C9—H9	119.1
C4—C3—H3	107.7	C10—C9—H9	119.1
C2—C3—H3	107.7	C11—C10—C9	119.33 (13)
C3—C4—C5	108.54 (11)	C11—C10—H10	120.3
C3—C4—H4A	110.0	C9—C10—H10	120.3
C5—C4—H4A	110.0	O3—C11—C10	123.94 (12)
C3—C4—H4B	110.0	O3—C11—C12	116.31 (12)
C5—C4—H4B	110.0	C10—C11—C12	119.75 (13)
H4A—C4—H4B	108.4	C13—C12—C11	120.02 (12)
C6—C5—C4	109.47 (10)	C13—C12—H12	120.0
C6—C5—H5A	109.8	C11—C12—H12	120.0
C4—C5—H5A	109.8	C12—C13—C8	121.16 (13)
C6—C5—H5B	109.8	C12—C13—H13	119.4
C4—C5—H5B	109.8	C8—C13—H13	119.4
H5A—C5—H5B	108.2	O3—C14—H14A	109.5
N1—C6—C5	112.35 (11)	O3—C14—H14B	109.5
N1—C6—H6A	109.1	H14A—C14—H14B	109.5
C5—C6—H6A	109.1	O3—C14—H14C	109.5
N1—C6—H6B	109.1	H14A—C14—H14C	109.5
C5—C6—H6B	109.1	H14B—C14—H14C	109.5
H6A—C6—H6B	107.9	C2—N1—C6	125.13 (11)
N1—C7—C8	112.04 (10)	C2—N1—C7	119.67 (11)
N1—C7—H7A	109.2	C6—N1—C7	114.78 (10)
C8—C7—H7A	109.2	C3—O2—H2	107.9 (12)
N1—C7—H7B	109.2	C11—O3—C14	117.08 (12)
			
O1—C2—C3—O2	−36.84 (16)	O3—C11—C12—C13	178.67 (13)
N1—C2—C3—O2	144.47 (12)	C10—C11—C12—C13	−1.3 (2)
O1—C2—C3—C4	−160.52 (12)	C11—C12—C13—C8	0.3 (2)
N1—C2—C3—C4	20.79 (15)	C9—C8—C13—C12	0.7 (2)
O2—C3—C4—C5	−174.31 (10)	C7—C8—C13—C12	−179.32 (12)
C2—C3—C4—C5	−50.17 (14)	O1—C2—N1—C6	176.47 (12)
C3—C4—C5—C6	64.96 (14)	C3—C2—N1—C6	−4.89 (17)
C4—C5—C6—N1	−48.71 (15)	O1—C2—N1—C7	4.33 (18)
N1—C7—C8—C9	−70.61 (15)	C3—C2—N1—C7	−177.02 (10)
N1—C7—C8—C13	109.37 (14)	C5—C6—N1—C2	19.25 (17)
C13—C8—C9—C10	−0.5 (2)	C5—C6—N1—C7	−168.28 (10)
C7—C8—C9—C10	179.46 (12)	C8—C7—N1—C2	98.18 (13)
C8—C9—C10—C11	−0.5 (2)	C8—C7—N1—C6	−74.74 (14)
C9—C10—C11—O3	−178.55 (12)	C10—C11—O3—C14	−1.19 (19)
C9—C10—C11—C12	1.5 (2)	C12—C11—O3—C14	178.80 (13)

### Hydrogen-bond geometry (Å, º)

**Table:**

*D*—H···*A*	*D*—H	H···*A*	*D*···*A*	*D*—H···*A*
O2—H2···O1^i^	0.96 (2)	1.84 (2)	2.7708 (16)	161.6 (19)
C6—H6*B*···O1^ii^	0.99	2.43	3.3142 (17)	148
C14—H14*B*···O2^iii^	0.98	2.52	3.449 (2)	158

Symmetry codes: (i) −*x*, −*y*, −*z*+1; (ii) *x*, −*y*+1/2, *z*−1/2; (iii) −*x*+1, −*y*, −*z*+1.
